# Improving Electrochemical Properties of Polypyrrole Coatings by Graphene Oxide and Carbon Nanotubes

**DOI:** 10.3390/nano10030507

**Published:** 2020-03-11

**Authors:** Nelly Maria Rosas-Laverde, Alina Pruna, David Busquets-Mataix

**Affiliations:** 1 Department of Materials and Mechanical Engineering, Universitat Politècnica de València, 46022 Valencia, Spain; nelly.rosas@epn.edu.ec; 2 Department of Materials, Escuela Politécnica Nacional, Quito 170517, Ecuador; 3 Center for Surface Science and Nanotechnology, Polytechnic University of Bucharest, 060042 Bucharest, Romania; 4 Institute of Materials Technology, Universitat Politècnica de València, 46022 Valencia, Spain; dbusquets@mcm.upv.es

**Keywords:** electrodeposition, graphene oxide, carbon nanotubes, polypyrrole, supercapacitor

## Abstract

Nanostructured polypyrrole coating was applied on carbon paper via simple dip-coating and electrochemical approach. Hybridization with nanocarbon materials (graphene oxide (GO) and multi-walled carbon nanotubes (MWCNTs)) and their effect as an anchoring hybrid layer for the growth of polypyrrole towards improving electrochemical properties are studied. The loading of each component and their *w/w* ratio were evaluated. Fourier transform infrared spectroscopy, field emission scanning electron microscopy, and Raman spectroscopy were employed to characterize the properties of the coatings. The electrochemical properties were investigated by cyclic voltammetry. The results indicated the electrodeposition of polypyrrole is enhanced by the addition of MWCNTs to the GO layer due to the formation of a hierarchical network. The electrochemical performance of the modified electrode was shown to be highly dependent on the employed *w/w* ratio, reaching a capacitance value of about 40 mF cm^−2^ for a carbon paper substrate modified with GO:MWCNT in a *w/w* ratio of 1:2.5 and PPy layer deposited by cyclic voltammetry for 30 cycles. The contribution to total stored charge was found to be primary from the inner capacitance component of about 95.5% contribution.

## 1. Introduction

In the last years, the worldwide interest for specific applications such as energy storage, sustainable and renewable energy and the fabrication of the related devices has greatly increased [1,2]. On these grounds, the clean energy storage emerged [3]. Supercapacitors, also known as ultracapacitors, are indicated as an alternative for energy storage [2,3,4], as they exhibit high power density, long cycling lifetime, fast rate of charge–discharge, short response time, good durability and safe operation [1,2,4,5,6]. The charge storage mechanism in supercapacitors is based on electrical double-layer capacitance (EDLC), which is related with adsorption and de-adsorption of ions in the electrode material/electrolyte interface, and on pseudocapacitance, which is related to reversible Faradaic redox reactions [5,7,8].

The carbon nanomaterials including graphene (G), reduced graphene oxide (rGO), carbon fibers (CF), carbon nanotubes (CNTs), porous carbon and carbon aerogels contribute with EDLC thanks to their high electrical conductivity, excellent mechanical strength and high surface area [1,7]. Graphene is a two-dimensional nanomaterial with sp^2^ hybridized carbons exhibiting high surface area, chemical stability, charge-carrier mobility, inherent flexibility and good transparency, which enable its use in different applications including biosensing, photovoltaics, batteries or fuel cells [2,9,10]. GO, an oxygen-decorated graphene derivative, is sometimes preferred instead of G due to its easy low-cost preparation and its chemical reactivity [10]. The cylindrical one-dimensional (1D) CNTs were also reported in energy storage applications by contributing with excellent electron-transport pathways, resilient skeleton and good percolation [11], as they exhibit large surface area, high electrical conductivity, chemical stability as well as an inherent high aspect ratio [4,8,12,13,14]. While carbon nanomaterials store charges only electrostatically at their surfaces which lowers their capacitance [3], a synergetic effect of G and CNTs for electrode materials in supercapacitors has been reported [15,16].

Materials including MnO_2_, Co_3_O_4_, NiO, Ni(OH)_2_, Co(OH)_2_ and conducting polymers have been indicated to show pseudocapacitive properties. Amongst the conducting polymers, polypyrrole (PPy) attracted increased interest in energy storage thanks to easy polymerization, high electrical conductivity [8], nontoxicity, high theoretical specific capacitance attributed to its good charge-storage performance in wide potential window [11,17], low cost, chemical and environmental stability [8,11,18]. Nevertheless, PPy may suffer from mechanical degradation during the intercalating/de-intercalating process which further result in limited cycling stability and high interfacial resistance [1,5,7,11,19,20].

Therefore, a common strategy to overcome these issues in fabricating supercapacitor electrodes considers the nanostructuring of conducting polymers and their hybridization with nanocarbons, so as to benefit from synergetic effects from the individual components [21,22,23,24,25,26,27,28,29,30,31,32]. A variety of synthesis methods has been employed to create such composites, including chemical polymerization and electrodeposition of conducting polymers. For example, the addition of GO sheets was shown to induce and influence greatly the fibrillar morphology of polymer and so, the specific capacitance of the nanocomposite was remarkably enhanced [33]. The formation of a continuous conducting network was reported upon the chemical polymerization of PPy onto GO-coated substrates, while the partial reduction of GO was indicated to be of paramount importance for the conductivity of the obtained electrode [34]. While the chemical polymerization can lead to aggregation and poor interconnection between conducting structures [35], the electropolymerization method was reported as the simplest and most efficient route to prepare PPy films on varying nanocarbons, including graphene [36], CNTs, and rGO/CNT papers [32,37], or for high-strength high-capacitance polyaniline/CNT-based composite [38]. Although the investigation parameters vary widely across the literature, the capacitance of GO/CNT/PPy-based electrodes was found to range from 29 to 108 mF cm^−2^ [8,19,39,40]. 

Carbon fiber (CF)-based substrates show interesting perspectives as a substrate for supercapacitors [16] thanks to its low cost [41], mechanical flexibility and strength [1,41], as well as its excellent conductivity [1,11,41], chemical stability [42] and electron/ion transport [43].

In this work, ternary composites based on GO, CNTs and PPy are applied by a simple electrochemical method for CF substrate modification. First the CF paper is chemically activated in order to improve its hydrophilicity and further it is modified with a GO coating. MWCNTs are employed to improve the GO coating conductivity, and to serve as spacers in order to form a porous GO/MWCNTs network. Thus, an improved specific surface area is obtained and the PPy growth is consequently enhanced [1], resulting in improved load transfer within the structure of the hybrid electrode and the capacitance, respectively [44]. A systematic investigation on the effect of the component loading as well as GO:CNT *w/w* ratio on the electrochemical properties for energy storage application is presented. The obtained results indicate a synergetic effect towards improving capacitance properties and the viability of the proposed approach for the fabrication of ternary composites with application in energy storage devices.

## 2. Materials and Methods

### 2.1. Materials

All chemicals (Alfa Aesar) were reagent grade and used as received. Exception was pyrrole monomer, which was distilled prior to use, and GO and multi-walled carbon nanotubes (MWCNTs), which were purchased from Sigma Aldrich and Cheaptubes, respectively. CF papers (TGP-H-060, Fuel Cell Store) were employed as substrate. Prior to use, the CF substrates were cleaned successively in acetone and isopropanol in ultrasonic bath for 10 min each. Finally, the substrates were dried under air.

### 2.2. Fabrication of Ternary PPy/rGO:CNT Composite

#### 2.2.1. Activation of CF Substrate

Given the hydrophobic property of carbon fibers and the composition of the CF substrate, it was shown that it requires surface pre-treatment prior to use [1]. Such treatment was indicated to improve not only the superficial properties of the CF substrate but also its conductivity and specific surface area, as well [41,45]. In this work, the activation process consisted in subjecting the substrate to 3 consecutive sonication steps in the varying solutions, namely 4 M NaOH solution for 30 min (followed by drying at 80 °C for 12 h), 2 M HCl for 30 min and distilled water for 30 min. Finally, the activated CF substrates were dried at 110 °C for 2 h.

#### 2.2.2. Modification of Activated CF Substrates with GO and MWCNTs

The CF substrates were modified with GO nanomaterials by dip-coating in the GO solution combined with bath sonication. Prior to use, the GO solution (0.5 mg/mL) was bath sonicated for 1 h. Varying substrate immersion duration and successive cycles were employed in order to analyze the effect of GO loading, namely condition (A) consisting in 1 min immersion in ultrasonic bath and five cycles while condition (B) considered 30 min immersion time and three cycles. After each immersion step the substrates were rinsed with distilled water and dried under airflow at 80 °C. The GO-modified activated CF substrates were denominated as GO*_x_*-modified substrates, where *x* is the condition employed for modification with GO.

In order to improve conductivity of GO coatings and provide with enhanced surface area, MWCNTs were added to the GO solution in varying GO:MWCNTs *w/w* ratio, namely 1:1 and 1:2.5. The rGO*_x_*:MWCNT*_y_*-modified substrates, where *y* corresponds to the employed ratio, were obtained in the same dip coating conditions as GO ones.

#### 2.2.3. Electrochemical Reduction of GO

The GO-modified CF substrates were further subjected to electrochemical reduction of GO by cyclic voltammetry (CV) method in a solution of 0.1 M KCl. The potential was swept between 0 and −1.4 V vs. Ag/AgCl reference electrode at 50 mV s^−1^ for 10 cycles without degassing. Finally, the obtained substrate was washed and dried in air.

#### 2.2.4. Deposition of PPy on rGO- and rGO:MWCNTs-modified CF Substrates

GO has been shown to exhibit nucleation sites for further deposition of conducting polymers [12]. Here, the GO-modified CF substrates were exploited for the electropolymerization of pyrrole (Py) by cyclic voltammetry from a solution of 0.1 M Py, 20 mM sodium dodecyl sulfate (SDS) and 0.05 M sodium p-toluenesulfonate (NapTS). The potential was swept in the range 0 to 1 V vs. Ag/AgCl at 50 mV s^−1^ for varying number of cycles between 10 and 30. Finally, the obtained substrates were rinsed with distilled water and dried at 60 °C for 12 h.

### 2.3. Characterization

All the electrochemical depositions were carried out by using a potensiostat (PGSTAT 302N AUTOLAB, Metrohm Autolab, Utrecht, The Netherlands) and NOVA 1.6 software. A conventional three-electrode electrochemical cell was assembled where the working electrode was the CF substrate, a Pt foil was employed as counter-electrode and the reference electrode was Ag/AgCl in saturated KCl. The obtained films were characterized in terms of structural and morphological characteristics by Fourier transform infrared spectroscopy (FTIR, Spectrum 100, Perkin Elmer, Waltham, MA, USA), field emission scanning electron microscopy (FESEM, Bruker working at 2Kv, Billerica, MA, USA) and Raman spectroscopy (LabRam HR UV (Horiba, Kyoto, Janpan) spectroscope using a He-Ne (632.8 nm) laser with a 1.6 cm^−1^ resolution). The specific surface area was determined by the Brunauer–Emmett–Teller (BET) equation and the pore size distribution of modified electrodes were determined by density functional theory (DFT) method [46], based on the nitrogen adsorption isotherm (77 K) (Quantachrome, Novatouch LX1, Boynton Beach, FL, USA). All samples were degassed at 65 °C for 24 h before analysis. The electrochemical measurements include CV tests in 0.5 M Na_2_SO_4_ solution in a potential range from −0.1 to 0.7 V vs. Ag/AgCl at varying scan rate between 2 and 100 mV s^−1^. The areal capacitance (*C_A_*) was calculated as reported elsewhere [7,8], by considering the potential window for cycling, the current, the scan rate and the apparent area, *A*, of the electrode (1 cm^2^) while the active material loading was about 1 mg cm^−2^. The cycling stability was evaluated in a potential window between −0.1 and 0.7 V vs. Ag/AgCl at a scan rate of 50 mV s^−1^ for 500 cycles.

## 3. Results

**Figure 1:**
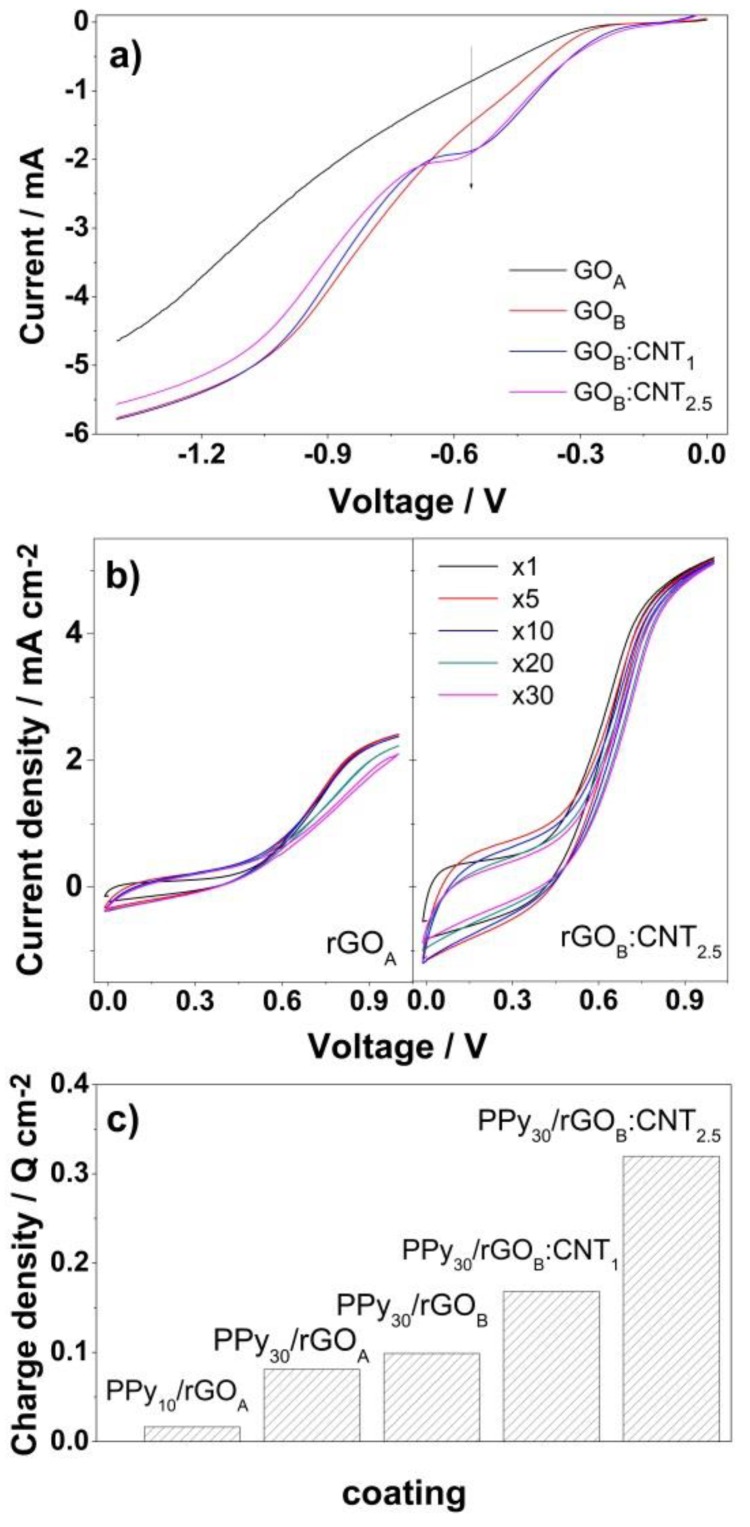
Cathodic scans of GO-modified substrates (V vs. Ag/AgCl) (**a**); voltammogram evolution for polypyrrole (PPy) electrodeposition with substrate and number of cycles (V vs. Ag/AgCl) (**b**); evolution of PPy deposition charge density as a function of loading cycle number (10 and 30) onto rGO_x_:CNT_y_ coatings (**c**).

Figure 1a depicts the first cathodic potential scans for the electroreduction of GO as a function of GO content and presence of MWCNTs. It is clear from Figure 1a that all types of GO-based coatings exhibit two characteristic reduction peaks at about −0.6 and −1.15 V vs. Ag/AgCl. It is observed that the peak current at −0.6 V increases when MWCNTs are present and the peaks slightly decreased with number of cycles which is indicating the reduction of GO nanomaterial.

The electropolymerization of Py monomer was analyzed as a function of cycle number and substrate nature. For exemplification, the CV curves for deposition of PPy at the surface of rGO_A_ and rGO_B_:CNT_2.5_-modified CF substrates are presented in Figure 1b. As it can be observed the oxidation of monomer at the surface of a rGO-modified substrate has the onset at about 0.6 V vs. Ag/AgCl [18]. The polymerization onset decreases with increasing rGO and MWCNTs loading, occurring at about 0.5 V vs. Ag/AgCl at the surface of the rGO_B_:CNT_2.5_-modified CF substrate, while the deposition current is greatly improved. The charge density corresponding to PPy deposition is further plotted in Figure 1c in order to analyze the growth of PPy at the surface of varying GO/MWCNTs coatings. It is shown that increasing PPy deposition cycles result in an increase of deposition charge. On the other hand, the increase in GO loading results in slightly improved PPy growth, further enhanced by the presence of MWCNTs.

**Figure 2:**
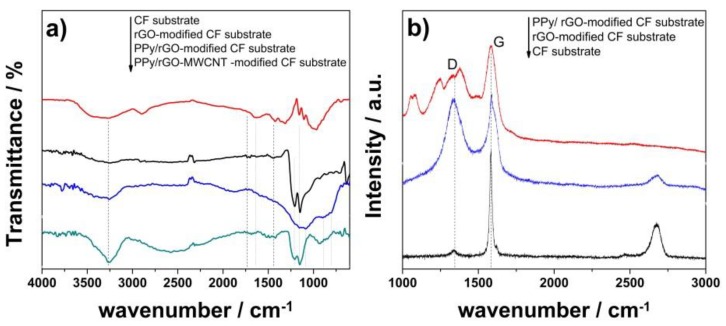
Fourier Transform InfraRed (FTIR) spectra of carbon fiber (CF) substrate before and upon coating with varying PPy/rGO:MWCNT coatings (**a**) and Raman spectra of activated CF substrate before and after coating with rGO and PPy/rGO, respectively (**b**).

The structure of composite coatings was analyzed by FTIR measurements, as depicted in Figure 2a. Despite low intensity of the peaks, the presence of rGO coating at the surface of CF substrate is indicated by the peaks located at 1720 cm^−1^ attributed to carbonyl/carboxyl C=O [47], 1629 cm^−1^ attributed to skeletal vibration of unoxidized graphitic domains (aromatic C=C) [48,49], 1438 cm^−1^ assigned to epoxy C–O, 1211 and 1152 cm^−1^ assigned to alkoxy C–O [50], and 1058 cm^−1^ attributed to carboxyl C–O groups [51,52]. The electropolymerization of pyrrole at the surface of rGO-modified substrate was indicated by the FTIR peak located at 1090 cm^−1^ which is attributed to the N–H in-plane deformation vibration and 1602 cm^−1^ assigned to vibrations of C=C in the PPy rings. At the same time, the polymerization of pyrrole is indicated by the peaks at 898 and 796 cm^−1^ [19,20]. Upon addition of MWCNTs to the rGO coating, the pyrrole presence is indicated by more intense peaks such as that located at 919 cm^−1^ or those ascribed to C–C and C–N stretching in the pyrrole ring and located at 1410 and 1520 cm^−1^ or the wide band at 3260 cm^−1^ attributed to N–H stretching vibration [53]. Although the bands for rGO and MWCNTs are weak in the composite coating and they may be overlapped with PPy [8], the typical FTIR characteristics are present for all components, thus confirming the fabrication of the composite coating.

The Raman spectra of modified CF substrates are further presented in Figure 2b. The activated CF substrate shows typical peaks for carbon fibers. The D band located at 1327 cm^−1^ is associated with disordered carbon vibrations, while the strong band at 1580 cm^−1^ is assigned to the G band associated with the E2g vibration of the graphitic structure. The second order peaks at 2464 cm^−1^ and an intense 2D band at 2676 cm^−1^ indicate the CF substrate is semi-graphitic [54,55]. By modification with rGO coating, the typical bands attributed to the defects and disorder in its structure and the sp2 hybridized carbons, namely D and G bands, appear at 1324 and 1601 cm^−1^ [56]. The rGO is effectively coating the substrate as the peaks of underlying substrate are much diminished, as indicated by the band at 2676 cm^−1^. A small shift appears in the position of the G peak, namely 1590 cm^−1^ in the Raman spectra corresponding to the PPy/rGO composite coating. The PPy presence is also indicated by the new band located at 1370 cm^−1^ attributed to the ring stretching and the one at 1492 cm^−1^ [57,58]. The peak intensity ratio I_D_/I_G_ for the rGO appears larger with respect to the composite.

**Figure 3:**
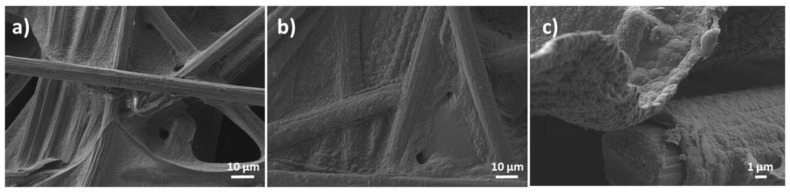
FE-SEM images of carbon fiber (CF) substrate before (**a**) and after modification with composite coating (**b**,**c**).

The morphology analysis revealed a network of carbon fibers randomly ordered for the activated CF substrate, as depicted in Figure 3a [1]. A continuous compact coating with globular microstructure, typical for the PPy [10] was obtained upon modification of CF substrate with the composite—see Figure 3b,c, in agreement with other studies on PPy electrosynthesis by CV methods [18]. The FESEM images were processed and resulted that PPy layer was deposited in about 500 nm thickness to maintain the porous network of the substrate.

**Figure 4:**
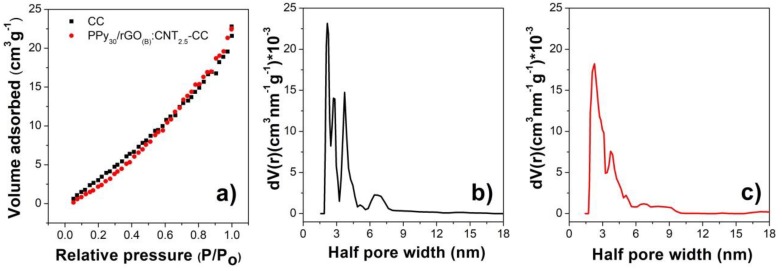
Nitrogen sorption analysis (**a**) of activated CF substrate before and after coating with PPy/rGO:MWCNT and corresponding pore size distribution by density functional theory (DFT) method (**b**,**c**).

The nitrogen adsorption isotherms and corresponding pore size distribution curves for the modified CF electrodes are further shown in Figure 4. The activated CF substrate exhibits a BET surface area of 35.67 m^2^ g^−1^ which increased upon modification with composite coatings, in agreement with other reports [1]. The composite with highest BET surface was the one with higher loading, namely PPy_30_/rGO_B_:MWCNT_2.5_, which exhibited a BET surface area of 103.0 m^2^ g^−1^. The half pore width distribution calculated by DFT method is shown in Figure 4b,c and indicates a half pore width of about 2.2 nm for PPy_30_/rGO_(B)_:MWCNT_2.5_-coated CF substrate [59].

**Figure 5:**
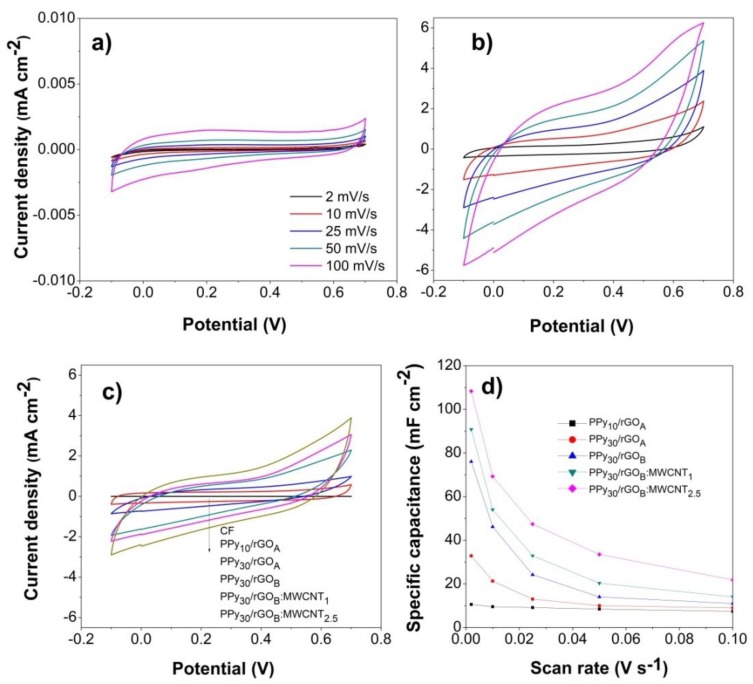
Cyclic voltammograms (CV) (V vs. Ag/AgCl) for CF substrate (**a**) and PPy_30_/rGO_(B)_:CNT_2.5_-modified one (**b**) with the scan rate (**c**); CV comparison for the modified substrates at 5 mV s^−1^ and (**d**) areal capacitance as a function of scan rate.

The electrochemical performance of the modified CF electrodes was evaluated by CV analysis and cycling stability tests. For exemplification, Figure 5a,b shows the voltammograms for the activated CF substrate before and upon modification with PPy_30_/rGO_(B)_:MWCNT_2.5_ coating at varying scan rate between 2 and 100 mV s^−1^. While an ohmic resistance appears in Figure 5b,c, which is attributed to the surface properties of the coatings, a clear increase in the electrochemical performance is observed upon modification with composite coating. The current increases linearly with the potential scan rate. For more insight into the effect of modification approach on the electrochemical performance, the voltammograms recorded at 25 mV s^−1^ are presented in Figure 5c. Higher loadings of each of components PPy, rGO and MWCNTs result in the enhancement of electrochemical performance of the electrode, where the maximum was reached for the PPy_30_/rGO_(B)_:MWCNT_2.5_-modified substrate with a *C_A_* value of 108.32 mF cm^−2^. Further, Figure 5d shows a decrease in areal capacitance with the scan rate. On the other hand, at high scan rates the storage process only occur at the electrode surface thus the interaction with the internal part where the most important capacitive processes take place is not permitted [60,61].

**Figure 6:**
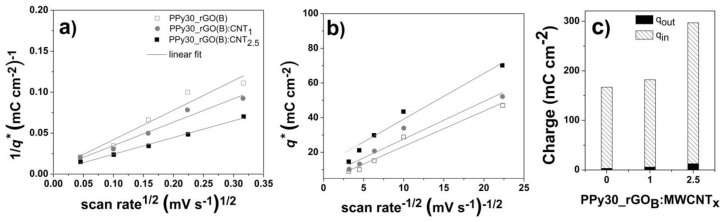
Trasatti plots (**a** and **b**) and the contribution to the total stored charge from the outer (*q_out_*) and inner (*q_in_*) charge components (**c**).

The hierarchical network produced between PPy and nanocarbons is considered for the improved electrolyte ion transport [1,16]. Moreover, the capacitive behavior was found to originate from both surface-controlled and diffusion-controlled counterparts, one resulting from outer electrochemical surface in direct contact with the electrolyte and the other related to the internal region of pores, dislocations, grain boundaries [62,63]. Thus, the Trasatti plots depicted in Figure 6 were employed to find the total charge (Figure 6a) and the outer charge (Figure 6b). The inner contribution to stored charge is then calculated from total and outer charge components and represented in Figure 6c.

**Figure 7:**
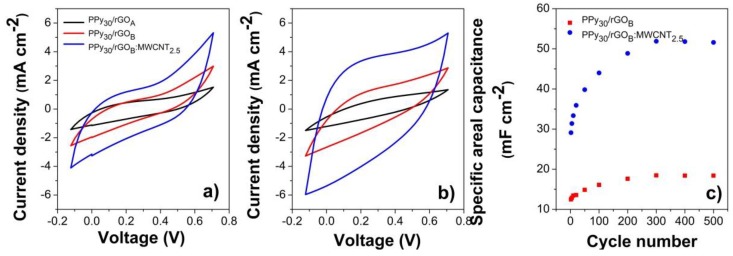
Evolution of CV (V vs. Ag/AgCl) curves of PPy_30_/rGO_A_, PPy_30_/rGO_B_ and PPy_30_/rGO_B_:CNT_2.5_ with cycling at 50 mV s^−1^: First cycle (**a**), 500th cycle (**b**) and areal capacitance as function of cycle number (**c**).

In order to further investigate the performance of the flexible electrodes, cyclic stability was tested as shown in Figure 7. As it can be seen in Figure 7a, a capacitive behavior results together with an ohmic resistance. However, as indicated in Figure 7b. The ohmic resistance is greatly diminished and the capacitive behavior improves. On the other hand, Figure 7c shows a capacitance increase with cycling, that is faster and in a higher gradient for the PPy_30_/rGO_B_:CNT_2.5_ coating with respect to the PPy_30_/rGO_B_ (1.96 increase with respect to 1.5). As the cycling results in Figure 7 show, the PPy_30_/rGO_B_ coating reaches stability after 200 cycles while upon the MWCNTs addition, the increase in capacitance reaches stability at about 500 cycles.

**Figure 8:**
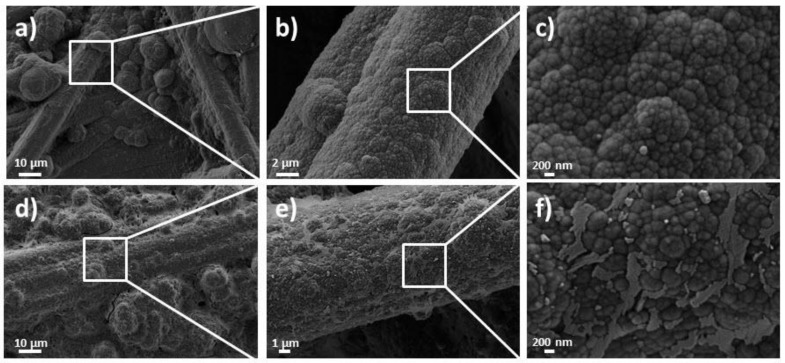
FE-SEM images of PPy_30_/rGO_B_:CNT_2.5_ at (**a**–**c**) zero cycles and (**d**–**f**) after 500 cycles.

Morphological changes are observed in the microstructure of the coating upon electrochemical cycling as it can be seen in Figure 8. The SEM images of the electrode recorded after 500 such cycles exhibit rougher edges that may significantly increase active surface area of electroactive material.

## 4. Discussion

The GO reduction peaks depicted in Figure 1a are associated with the reduction of oxygen functional groups decorating the GO nanomaterial, in agreement with other reports [9]. The reduction peaks become more evident with the GO loading due to the presence of an increasing number of oxygen groups that would be reduced. On the other hand, the increase in the peak current at −0.6 V vs. Ag/AgCl with the presence of MWCNTs could be attributed to spacer role of MWCNTs aiding in an enhanced exposure of oxygen groups between the GO layers to be reduced.

Regarding the growth of PPy onto the hybrid coating, the decrease in the polymerization onset with increasing rGO and MWCNTs loading is attributed to the enhanced number of residual groups in rGO that serve as nucleation sites for the electropolymerization of Py [19], and to the improved conductivity conferred by the presence of MWCNTs, respectively. The obtained PPy deposition charge gradient indicates a strong interaction between the rGO layer and the formed PPy, which results in a synergetic effect for the physical and chemical interactions at the CF/PPy interface [10]. The negligible increase in PPy growth with GO is due to the extent of removal of oxygen groups from a thicker GO coating. This effect is further confirmed by addition of MWCNTs which results in a marked improvement in PPy deposition due to enhanced conductivity and spacer function of MWCNTs in the GO/MWCNT network [16].

The FTIR results confirmed the modification with rGO nanomaterial of the electrode surface. The enhanced peaks appeared upon the addition of MWCNTs to the rGO coating are attributed to the improved conductivity of the electrode which leads to the improved electropolymerization of pyrrole. The modification of the CF substrate with carbon nanomaterial and polypyrrole was further confirmed by Raman results. The small shift in the position of the G peak in the Raman spectra corresponding to the PPy/rGO composite coating is indicative of marked coupling and charge transfer between the PPy and rGO [64]. The larger value of I_D_/I_G_ for the rGO with respect to the composite is indicative of less defective carbon network in the composite which is attributed to the interaction between PPy and rGO layer.

The morphology images in Figure 3b,c revealed a continuous compact coating with globular microstructure of the PPy on modified CF substrate with the composite which suggests a strong interaction between the rGO and PPy attributed to the active nucleation sites offered by rGO for PPy growth [44] and the CF substrate. Such interaction and interconnection are desired for improving electrode performance [11].

The electrochemical results show a quasi-rectangular shape of the activated CF substrate which indicates a capacitive behavior [8,65], while the modified one exhibits a pseudocapacitive one [1,5,66]. The increase in the electrochemical performance upon modification of CF substrate with composite coating is attributed to the interaction and network formed between the components rGO, MWCNTs and PPy, which contribute to the capacitive properties [19,42,65]. The linear current increase with the potential scan rate is indicative of a diffusion-controlled process. Nevertheless, the combination of capacitive and intercalation effects towards the total charge was observed to depend on the applied potential.

The evolution of the electrochemical performance with increase in the component loading (PPy, rGO and MWCNTs) showed an enhancement which is attributed to improved surface area and conductivity due to the presence of rGO and MWCNTs, respectively, and the interaction with PPy network that could reduce the charge transport pathways [42]. This is supported by the contribution to the stored charged, as indicated by the values of outer charge and total charge obtained from Trasatti plots in Figure 6. It is clearly observed that the major contribution to the stored charge is from the inner charge related to the pores and network induced by the addition of MWCNTs to the GO coating, which further results in enhanced interaction with the PPy towards reducing the charge transport paths.

The estimated results in Figure 6c suggesting the dominance of redox reactions by almost 96% inner charge contribution to the total stored charge are further supported by the cycling results in Figure 7. Thus, the slight shift of the redox peaks in Figure 7a,b upon cycling is attributed to penetration of electrolyte ions into the porous coatings. It is considered that the surface of the coating is not fully wetted at the initial stage which delays the electrolyte ions penetration and an increase in active surface area is achieved upon cycling. However, other reports suggested the electrolyte ions may intercalate upon cycling and form active centers for further redox reactions [67].

The capacitance increases upon cycling as observed in Figure 7c, which may be related to a self-activation process of the modified electrode due to an increase in active sites through an increase in active surface area. The capacitance reached stability upon cycling after about 300 cycles, as reported for other electrodes such as CNT/MnMoO_4_ [65], CNT-GO/PPy [8], manganese oxide nanorod arrays [68] and NiCo_2_S_4_@NiO NWAs [69], which reached the stability only after 300, 2000, 1500 and 5000 cycles, respectively.

The rougher morphology obtained for the modified substrate upon cycling is in agreement with other studies showing electron transfer-assisted nanostructuring of the active material with continuous electrochemical cycling, that cause an improvement in the active surface area [65]. The morphology results confirmed the MWCNTs addition to a PPY/rGO composite aid in the formation of hierarchical network by working as a spacer between GO nanosheets, which results in a faster and facile electron transfer across the electrode/electrolyte interface [65].

The capacitance values obtained by modifying the activated CF substrate with PPy/rGO:MWCNTs composite coatings are in line with other reported studies [8,19], which indicate the viability of the approach for the fabrication of porous coatings with applicability in energy storage.

## 5. Conclusions

Carbon paper was coated with a hierarchical network of hybrid coating consisting in GO, CNTs and PPy by simple dip-coating and electrochemical methods. The GO served as an anchoring layer for the growth of polypyrrole upon a chemical reduction by cyclic voltammetry. The addition of CNTs to the GO layer was investigated. The results indicated the formation of a ternary hybrid coating, where the growth of PPy was enhanced with the addition of CNTs to the GO layer. The electrochemical results indicated a synergetic effect of the GO, CNTs and PPY towards a good capacitive behavior, the highest loading of GO and a 1:2.5 *w/w* ratio GO:MWCNTs allowing the formation of an electrode with high potential for electrochemical capacitors. The effect of the MWCNTs addition is confirmed by the major contribution from the inner surface to the total stored charge. The areal capacitance decreases with the scan rate which is attributed to the ion diffusion and charge transfer processes occurring both on the internal active surface and the external part, which result in high CA values at low scan rates.
